# Bafilomycin A1 enhances NLRP3 inflammasome activation in human monocytes independent of lysosomal acidification

**DOI:** 10.1111/febs.15619

**Published:** 2020-11-21

**Authors:** Shi Yu, Jack Green, Rose Wellens, Gloria Lopez‐Castejon, David Brough

**Affiliations:** ^1^ Division of Neuroscience and Experimental Psychology School of Biological Sciences Faculty of Biology, Medicine and Health Manchester Academic Health Science Centre University of Manchester UK; ^2^ Lydia Becker Institute of Immunology and Inflammation University of Manchester UK; ^3^ Manchester Collaborative Centre for Inflammation Research (MCCIR) Division of Infection Immunity & Respiratory Medicine School of Biological Sciences Faculty of Biology, Medicine and Health Manchester Academic Health Science Centre University of Manchester UK

**Keywords:** alternative NLRP3 inflammasome, caspase 1, cytokine, immune cell, immunology, inflammasome, inflammation, interleukin 1, monocyte, NLRP3

## Abstract

The release of interleukin (IL)‐1β from primary human monocytes in response to extracellular LPS occurs through the NACHT, LRR and PYD domains‐containing protein 3 (NLRP3) inflammasome. In primary monocytes, in response to LPS, NLRP3 inflammasome activation is characterized by an independence of K^+^ efflux and ASC speck formation and has been termed the ‘alternative’ pathway. Here, we report that pharmacological inhibition of V‐ATPase with bafilomycin A1 exacerbated LPS‐induced NLRP3 inflammasome activation in primary human monocytes. Inhibition of V‐ATPase in the presence of extracellular LPS led to NLRP3‐dependent, K^+^ efflux‐independent, ASC oligomerization and caspase‐1 activation. Although V‐ATPases are required for lysosomal acidification, we found that acidic lysosomal pH and protease activity were dispensable for this altered response, suggesting that V‐ATPase inhibition triggered alternative signalling events. Therefore, V‐ATPases may serve additional roles during NLRP3 inflammasome activation in primary human monocytes.

## Graphical abstract

The NLRP3 inflammasome drives inflammation in response to tissue damage and infection by promoting the processing and release of IL‐1β. Human monocytes have an alternative pathway of NLRP3 inflammasome activation in response to lipopolysaccharide alone. Here, we report that the vacuolar ATPase, a lysosomal proton pump, is a regulator of the alternative NLRP3 response.

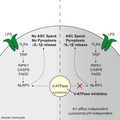

**Abbreviations**
ASC: apoptosis‐associated speck‐like protein containing a CARD
CARD: caspase activation and recruitment domain
GSDMD: gasdermin D
IL: interleukin
LPS: lipopolysaccharide
NLR: NOD‐like receptor
NLRP3: NACHT, LRR and PYD domains‐containing protein 3
PBMCs: peripheral blood mononuclear cells
PGN: peptidoglycan
SAA: serum amyloid A
TLR: Toll‐like receptor
V‐ATPase: vacuolar ATPase

## Introduction

Inflammasomes serve as the major site for activation for caspase‐1 and pro‐inflammatory cytokines IL‐1β and IL‐18. Understanding the regulation of IL‐1β is particularly important as its dysregulation is strongly associated with inflammatory diseases [1]. There are a number of different types of inflammasome. A commonly studied inflammasome and one known to contribute to inflammation within multiple diseases is formed by the NOD‐like receptor (NLR) family member NLRP3 (NACHT, LRR and PYD domains‐containing protein 3). Due to its association with diverse diseases, there is major interest in understanding the regulation of the NLRP3‐IL‐1β pathway [2]. There are several suggested pathways for the activation of NLRP3, the best studied of which is termed the ‘canonical’ pathway, reported mainly in macrophages, and which requires two signals. The initial stimulus is a priming signal, where pathogen‐associated molecular patterns (PAMPs), or damage‐associated molecular patterns (DAMPs), are sensed by PRRs, which then transduce an intracellular signal resulting in increased expression of NLRP3, and an inactive precursor pro‐IL‐1β. The second stage of canonical NLRP3 inflammasome activation occurs when a primed cell receives an additional stimulus, often causing K^+^ efflux from the cell, which triggers a change in the conformation of NLRP3 that allows it to bind to an adaptor protein called apoptosis‐associated speck‐like protein containing a CARD (ASC). The NLRP3‐ASC interaction drives the polymerization of ASC into what is known as the ASC speck. The cysteine protease caspase‐1 is then recruited to, and activated at, the ASC speck. The whole complex is known as the NLRP3 inflammasome [2]. Activated caspase‐1 at the inflammasome complex then cleaves pro‐IL‐1β to a mature IL‐1β molecule, which is then secreted from the cell via gasdermin D (GSDMD) pores to induce an inflammatory response [3, 4]. GSDMD pores can also drive pyroptosis which leads to the release of inflammasome complexes and additional DAMPs [5, 6].

In contrast to macrophages, in primary human monocytes a singular PAMP stimulus, for example extracellular lipopolysaccharide (LPS), is sufficient to trigger NLRP3‐dependent activation of caspase‐1 and IL‐1β secretion [7, 8, 9]. In human monocytes, LPS‐induced IL‐1β release occurs with no detectable ASC oligomerization, is independent of K^+^ efflux, and has been termed the ‘alternative’ NLRP3 inflammasome [8]. LPS‐induced NLRP3 inflammasome activation in human monocytes occurs in the absence of cell death and can further respond to NLRP3 agonists [8], unlike noncanonical NLRP3 inflammasome activation driven by intracellular LPS, caspase‐4, and GSDMD [10]. It is important to further our understanding of the NLRP3 inflammasome in human blood, in order to explore disease models associated with acute infection such as sepsis or chronic inflammatory conditions such as atherosclerosis. Here we discovered that vacuolar ATPase (V‐ATPase) inhibitors enhanced activity of the NLRP3 inflammasome in primary human monocytes, and the effect was independent of an effect on lysosomal pH. The V‐ATPase is a multi‐subunit protein complex that regulates many cellular processes [11]. Well known for a regulatory role on lysosomal pH and degradation of autophagic cargo, the V‐ATPase has additional roles in regulating protein sorting and cellular homeostasis [12, 13]. Thus understanding V‐ATPase‐dependent regulation of NLRP3 will lead to new insights into the signalling pathways coordinating inflammatory responses.

## Results

### Inhibition of V‐ATPase regulates ASC oligomerization in primary human monocytes

**Fig. 1:**
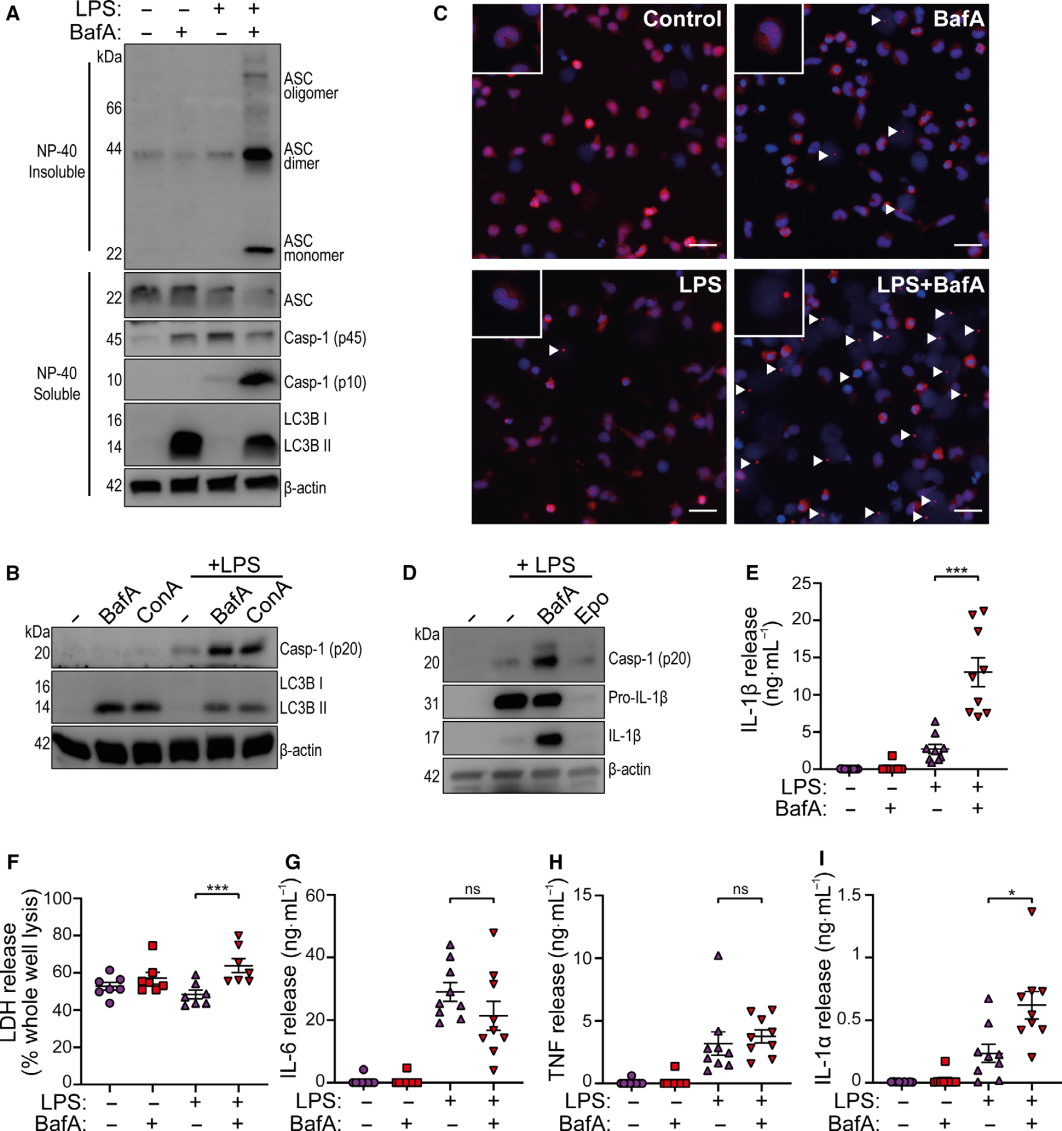
The V‐ATPase regulates the NLRP3 inflammasome in primary CD14+ monocytes. (A) Western blots of DSS cross‐linked Nonidet P‐40 (NP‐40) insoluble ASC oligomers and NP‐40 soluble ASC, pro‐caspase‐1 (p45), mature caspase‐1 (p10), and lipidated LC3B, detected from human CD14+ monocyte total cell lysates stimulated with LPS (1 µg·mL^−1^) plus or minus bafilomycin A1 (BafA, 100 nm) for 18 h (*n* = 6). (B) Western blots of mature caspase‐1 (p20) and LC3B detected from CD14+ monocyte total lysates (cell lysates + supernatants) stimulated with BafA (100 nm) or concanamycin A (ConA, 100 nm) plus or minus LPS (1 µg·mL^−1^) for 18 h (*n* = 4). (C) Representative immunofluorescence images of CD14 + monocytes immunostained for ASC in AlexaFluor 594 (red), with a DAPI (blue) nuclear co‐stain after stimulation with LPS (1 µg·mL^−1^) plus and minus BafA (100 nm) for 18 h (scale bar is 25 µm, arrow heads denote ASC specks) (*n* = 4). (D) Western blot of mature caspase‐1 (p20), pro‐IL‐1β (p31), mature IL‐1β (p17) and β‐actin, from human CD14+ monocyte total cell lysate stimulated with LPS (1 μg·mL^−1^) in the presence of V‐ATPase inhibitor bafilomycin A1 (BafA, 100 nm) or chymotrypsin inhibitor epoxomicin (Epo, 100 nm) (*n* = 3). (E) IL‐1β release and (F) cell death measured by ELISA and LDH assay respectively from the supernatants of CD14+ monocytes stimulated with LPS (1 µg·mL^−1^) and/or BafA (100 nm) for 18 h (*n* = 9). (G) IL‐6, (H) TNF and (I) IL‐1α release measured by ELISA, from human CD14+ monocyte supernatants stimulated with LPS (1 µg·mL^−1^) in the presence or absence of BafA, (100 nm) for 18 h (*n* = 9). *P < 0.05, ***P < 0.001; ns, not significantly different determined by a one‐way ANOVA with Sidak’s *post* *hoc* comparison. Values shown are the mean ± SEM.

Primary human monocytes were collected by positive selection for cell surface CD14 from peripheral blood mononuclear cells (PBMCs) obtained from healthy human donors. Fresh monocytes were immediately challenged with LPS (1 µg·mL^−1^, 18 h). Western blotting of the nonidet P‐40 (NP‐40)‐insoluble fraction cross‐linked with disuccinimidyl suberate (DSS) showed that LPS alone caused minimal ASC oligomerization (Fig. 1A). Western blotting of the soluble fraction confirmed that LPS alone activated caspase‐1, as evidenced by the presence of the caspase‐1 p10 subunit (Fig. 1A). Treatment with a V‐ATPase inhibitor bafilomycin A1 (100 nm, BafA) [14] at the same time as LPS, caused extensive ASC oligomerization and increased caspase‐1 activation, while addition of bafilomycin A1 alone did not activate caspase‐1 (Fig. 1A). Bafilomycin A1 is an inhibitor of lysosomal acidification, where it is known to prevent the degradation of autophagic vacuoles [15, 16]. Indeed, increased levels of the lipidated microtubule‐associated proteins 1A/1B light chain 3B (LC3B), a marker of the autophagosome membrane [17], were observed after bafilomycin A1 treatment indicating that lysosomal degradation was inhibited (Fig. 1A). The importance of the V‐ATPase for the regulation of the NLRP3 pathway was confirmed by treating primary human monocytes with LPS, plus and minus bafilomycin A1, and another specific inhibitor of the V‐ATPase, concanamycin A (100 nm, ConA) [18]. Both bafilomycin A1 and concanamycin A increased levels of active caspase‐1 p20 and lipidated LC3B in the presence of LPS (Fig. 1B). The increased ASC oligomerization observed by western blot above was mirrored by immunocytochemistry for ASC where ASC specks were revealed by LPS and bafilomycin A1 co‐treatment of monocytes using wide‐field fluorescence microscopy (Fig. 1C). To rule out proteasomal degradation of NLRP3 accounting for changes in caspase‐1 activation we used epoxomicin, a proteasomal chymotrypsin activity inhibitor, which had no effect on LPS‐induced caspase‐1 activation, but strongly inhibited pro‐IL‐1β expression (Fig. 1D). As a result of exacerbated inflammasome ASC speck formation and caspase‐1 activation, LPS and bafilomycin A1 co‐treatment caused a significant increase in IL‐1β secretion from human CD14 monocytes compared to LPS alone (Fig. 1E). Furthermore LPS and bafilomycin A1 together caused a significant increase in cell death as measured by lactate dehydrogenase (LDH) release (Fig. 1F). LPS‐induced release of IL‐6, and TNF, were not affected by bafilomycin A1, although bafilomycin A1 did induce a significant increase in the release of IL‐1α (Fig. 1G‐I). IL‐1α can also be regulated by the NLRP3 inflammasome [19]. These data suggest that the effects of bafilomycin A1 could be specific for the NLRP3 inflammasome and unrelated to the release of other cytokines.

### Effects of V‐ATPase inhibition are NLRP3 dependent

**Fig. 2:**
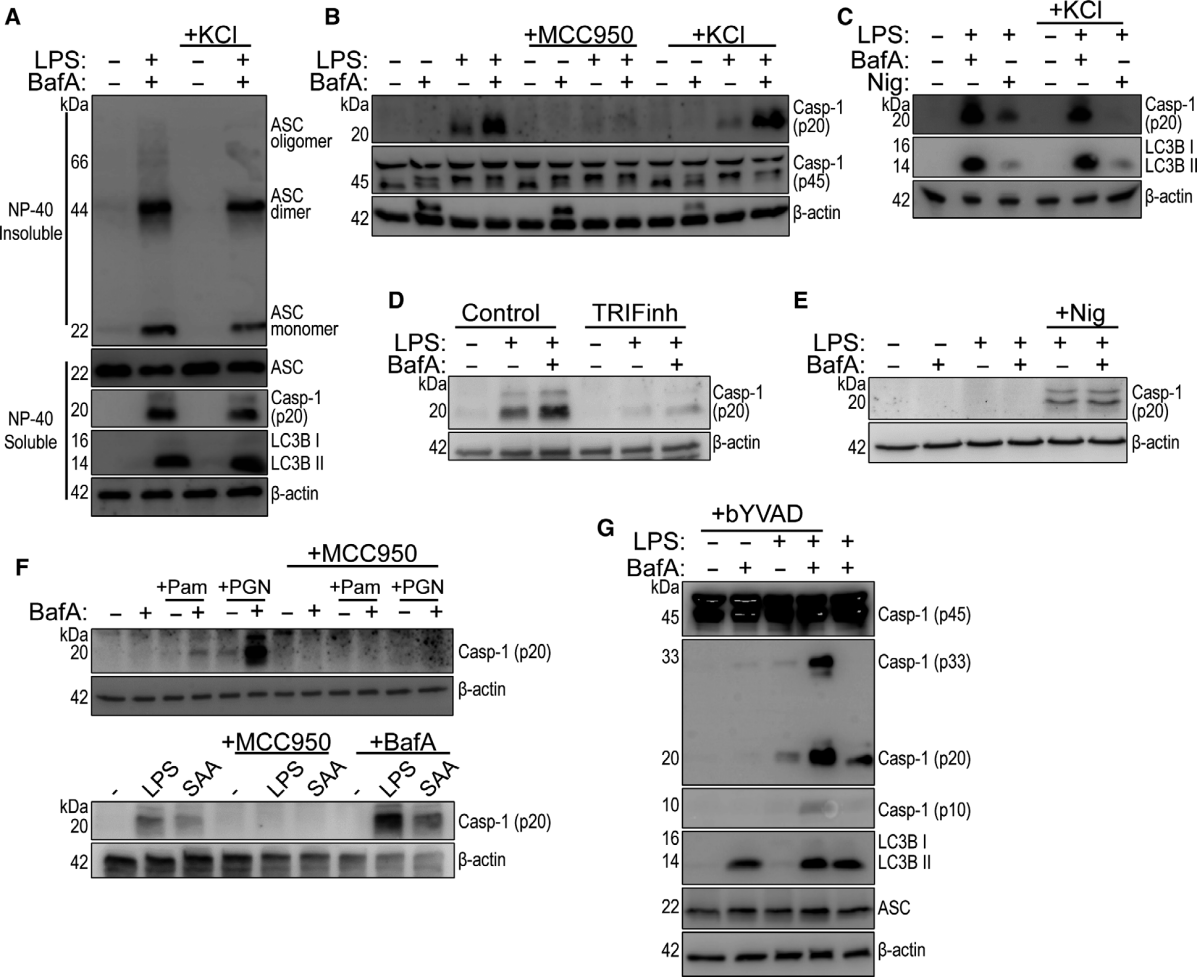
NLRP3 dependence of the V‐ATPase effect in monocytes. (A) Western blots of DSS cross‐linked NP‐40 insoluble ASC oligomers and NP‐40 soluble ASC, caspase‐1 (p20), and LC3B from CD14+ monocyte total cell lysates stimulated with LPS (1 µg·mL^−1^) plus and minus bafilomycin A1 (BafA, 100 nm) and extracellular KCl (20 mm) for 18 h (*n* = 4). (B) Western blot of mature caspase‐1 (p20) from CD14 + monocyte total cell lysates stimulated with LPS (1 µg·mL^−1^) plus and minus BafA (100 nm) supplemented with MCC950 (5 µm) or extracellular KCl (20 mm) (*n* = 3). (C) Western blot of mature caspase‐1 (p20) from CD14+ monocyte total cell lysate stimulated with LPS (1 µg·mL^−1^) plus and minus BafA (100 nm) or nigericin (Nig, 10 µm) supplemented with extracellular KCl (20 mm) for 18 h. Nigericin was added at the last hour of stimulation (*n* = 2). (D) Western blot of caspase‐1 (p20) from CD14 + monocyte total cell lysate treated with control peptide or TRIF blocking peptide (TRIFinh, 50 µm) for 2 h, before stimulation with LPS (1 µg·mL^−1^) plus and minus BafA (100 nm) for 18 h (*n* = 4). (E) Western blot of caspase‐1 (p20) from naïve or LPS‐primed (1 µg·mL^−1^, 4 h) THP‐1 monocyte total cell lysates preincubated with or without BafA (100 nm, 15 min) before addition of nigericin (10 µm, 1 h) (*n* = 4). (F) Upper panel: Western blot of mature caspase‐1 (p20), and β‐actin, from CD14 + monocyte total cell lysate stimulated with Pam3CSK4 (Pam, 10 μg·mL^−1^), or PGN from *S*. *aureus* (PGN, 20 μg·mL^−1^) plus or minus bafilomycin A1 (BafA, 100 nm) and/or MCC950 (10 μm) for 18 h (*n* = 3). Lower panel: Western blot of mature caspase‐1 (p20), and β‐actin, from CD14+ monocyte total cell lysate stimulated with LPS (1 μg·mL^−1^) or SAA (5 μg·mL^−1^) plus or minus MCC950 (10 μm) and BafA (100 nm) for 18 h (*n* = 2). (G) Western blot of caspase‐1 p45, p33, p20 and p10 species, and lipidated LC3B detected from CD14+ monocyte total lysates stimulated with LPS (1 µg·mL^−1^) plus and minus BafA, (100 nm) or biotin‐YVAD‐cmk (bVAD, 50 µm) for 18 h (*n* = 5).

To confirm that the increased ASC oligomerization observed in the presence of LPS and bafilomycin A1 was due to NLRP3, we performed the above experiment in CD14+ monocytes with LPS plus and minus raised extracellular K^+^ (+20 mm KCl) which is known to inhibit the canonical NLRP3 pathway [20]. There was no difference in ASC oligomerization, caspase‐1 activation, or LC3B lipidation between LPS and bafilomycin A1 treated cells in the absence or presence of high K^+^ (Fig. 2A). However, the NLRP3 inflammasome inhibitor MCC950 [21] inhibited the appearance of active caspase‐1 in response to LPS, and LPS plus bafilomycin A1, in CD14+ monocytes confirming that indeed we were manipulating the NLRP3 pathway (Fig. 2B). Raised extracellular K^+^ inhibited nigericin‐induced NLRP3 activation in LPS treated CD14+ primary monocytes (Fig. 2C), suggesting that bafilomycin A1 may be enhancing the alternative NLRP3 pathway. The alternative NLRP3 inflammasome, unlike the canonical, depends on the Toll‐like receptor (TLR)‐adaptor protein TRIF [8]. Pretreatment of CD14+ monocytes with a TRIF inhibitory peptide (TRIFinh, 50 µm) inhibited caspase‐1 activation in response to LPS treatment (Fig. 2D). Moreover, the TRIF inhibitory peptide also inhibited caspase‐1 activation in response to LPS and bafilomycin A1 treatment (Fig. 2D). Bafilomycin A1 addition after LPS pretreatment (1 µg·mL^−1^, 4 h) did not enhance nigericin‐induced caspase‐1 activation in THP‐1 monocytes (Fig. 2E), suggesting that bafilomycin A1 was selectively enhancing the alternative NLRP3 pathway. Bafilomycin A1 also enhanced MCC950‐sensitive caspase‐1 activation induced by previously described TLR2 agonists [9], such as the synthetic Pam3CSK4 (10 µg·mL^−1^), peptidoglycan (PGN) from *Staphylococcus aureus* (PGN, 20 µg·mL^−1^), and endogenous TLR4 agonist human serum amyloid A (SAA, 5 µg·mL^−1^) in primary human monocytes (Fig. 2F) showing that the effects of V‐ATPase inhibition were not limited to LPS. Caspase‐1 is produced as a 45 kDa pro‐form. Activation of pro‐caspase‐1 generates firstly a p33 caspase‐1 species which is followed by caspase activation and recruitment domain (CARD) domain linker cleavage leading to p20 and p10 heterodimers [22]. We repeated the experiment with LPS and bafilomycin A1 in human monocytes in the presence of a cell‐permeable irreversible caspase‐1‐specific probe, biotin‐YVAD‐cmk (bYVAD, 50 μm). After NLRP3 inflammasome stimulation with LPS, the bYVAD trapped more caspase‐1 in its active p33 form in the bafilomycin A1 and LPS treated cells, compared to LPS alone (Fig. 2G). These data suggest caspase‐1 activity is enhanced by the presence of the ASC speck.

**Fig. 3:**
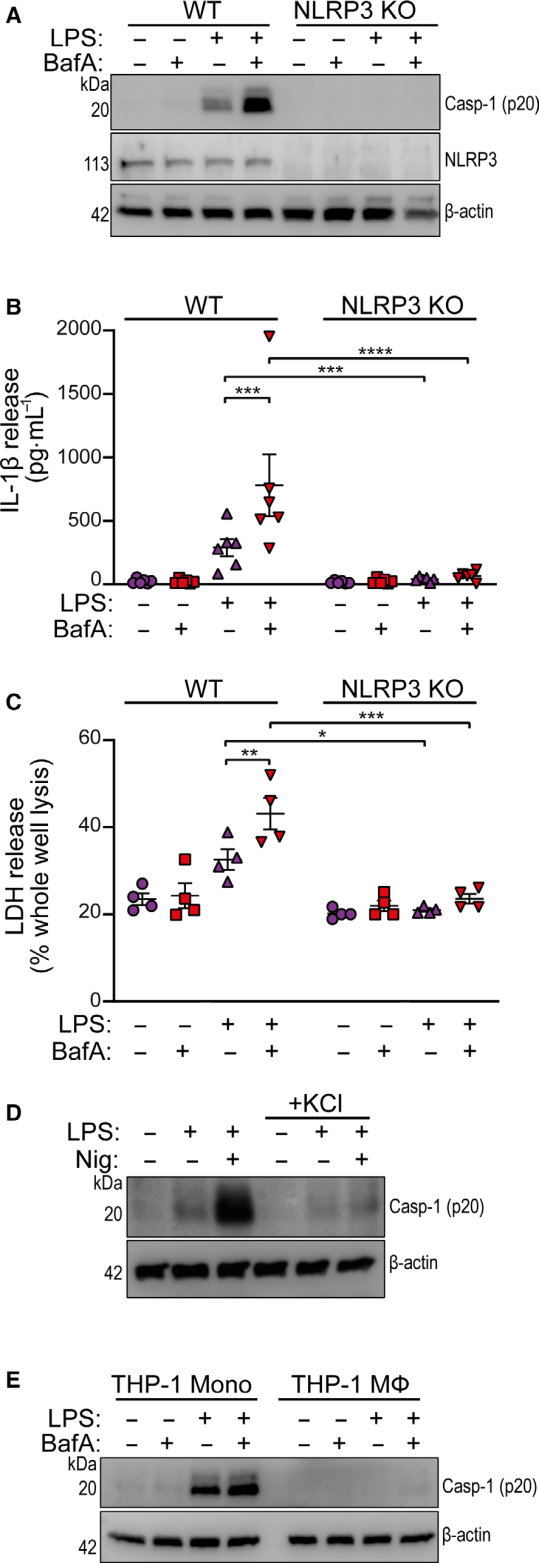
V‐ATPase inhibition enhances NLRP3‐dependent caspase‐1 maturation in THP‐1 cells. (A) Western blot of mature caspase‐1 (p20), NLRP3, and β‐actin from undifferentiated WT or NLRP3 KO THP‐1 total cell lysates stimulated with LPS (1 µg·mL^−1^) plus and minus bafilomycin A1 (BafA, 100 nm) for 18 h (*n* = 3). (B) IL‐1β release measured by ELISA, and (C) cell death measured by LDH release, from WT and NLRP3 KO THP‐1 cell supernatants stimulated with LPS (1 µg·mL^−1^) plus and minus BafA (100 nm) for 18 h (*n* = 6 and *n* = 4 respectively). (D) Western blot of mature caspase‐1 (p20) and β‐actin from undifferentiated WT THP‐1 cells stimulated with LPS (1 µg·mL^−1^) plus and minus nigericin (Nig, 10 µm) in the absence or presence of extracellular KCl (20 mm) for 18 h. Nigericin was added at the last hour of stimulation (*n* = 3). (E) Western blot of mature caspase‐1 (p20) and β‐actin from undifferentiated or PMA (250 nm, 16 h) differentiated WT THP‐1 total cell lysates stimulated with LPS (1 µg·mL^−1^) plus and minus BafA (100 nm) for 18 h (*n* = 2). *P < 0.05, **P < 0.01, ***P < 0.001, ****P < 0.0001 determined by a two‐way ANOVA with Sidak’s *post* *hoc* comparison. Values shown are the mean ± SEM.

We were also able to use THP‐1 monocytes to study the NLRP3 dependence of V‐ATPase inhibition. Undifferentiated WT and NLRP3 knockout (NLRP3 KO) THP‐1 cells were treated with bafilomycin A1, LPS, or LPS and bafilomycin A1. In WT, but not NLRP3 KO THP‐1 cells, LPS alone induced robust caspase‐1 activation and this was further enhanced by the presence of bafilomycin A1 (Fig. 3A). Similar to the primary monocytes, IL‐1β release was significantly enhanced in WT THP‐1 cells treated with bafilomycin A1 and LPS, compared to LPS alone, and this was absent in NLRP3 KO cells (Fig. 3B). The enhanced cell death caused by LPS and bafilomycin A1 treatment was also NLRP3‐dependent as LDH release was inhibited in treated NLRP3 KO THP‐1 cells (Fig. 3C). LPS‐induced caspase‐1 activation in undifferentiated THP‐1 cells was not inhibited by addition of 20 mm K^+^, whereas increased caspase‐1 activity triggered by nigericin treatment (10 µm) was inhibited by high extracellular K^+^ (Fig. 3D). LPS alone, or LPS plus bafilomycin A1, did not activate caspase‐1 in phorbol myristate acetate (PMA) differentiated THP‐1 macrophages (Fig. 3E).

### V‐ATPase dependent effects on NLRP3 are independent of lysosomal pH

**Fig. 4:**
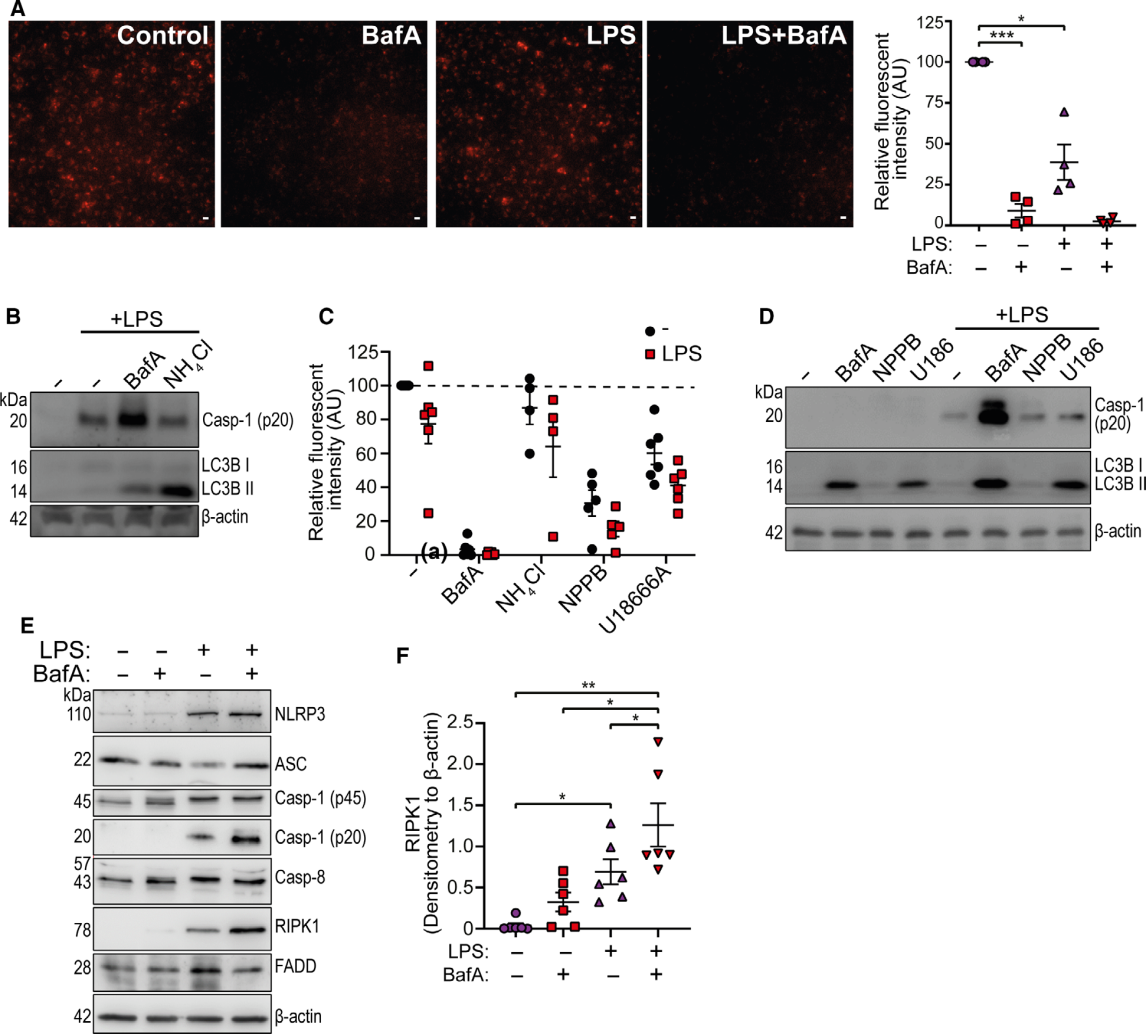
The effects of V‐ATPase inhibition on NLRP3 are independent of lysosomal pH. (A) Representative images and relative fluorescent intensity quantification (emission at 620 nm) from CD14 + monocytes treated with LPS (1 µg·mL^−1^) or bafilomycin A1 (BafA, 100 nm) in the presence of 10 µg·mL^−1^ DQ‐BSA Red for 18 h (Scale bar 25 µm, *n* = 4). (B) Western blot of mature caspase‐1 (p20), lipidated LC3B, and β‐actin from CD14+ monocyte total cell lysate stimulated with LPS (1 µg·mL^−1^) plus BafA (100 nm) or NH_4_Cl (5 mm) for 18 h (*n* = 4). (C) Relative fluorescence intensity of CD14+ monocytes treated with or without LPS plus NH_4_Cl (5 mm), NPPB (50 μm), U18666A (20 μm) in the presence of 10 µg·mL^−1^ DQ‐BSA Red for 18 h (*n* = 6). (D) Western blot of mature caspase‐1 (p20), lipidated LC3B, and β‐actin from CD14 + monocyte total cell lysate stimulated with BafA (100 nm), NPPB (50 μm) or U18666A (U186, 20 μm) for 18 h (*n* = 3). (E) Western blot of NLRP3, ASC, pro‐caspase‐1 (p45), mature caspase‐1 (p20), caspase‐8, RIPK1 and FADD from CD14+ monocyte total cell lysate stimulated with LPS (1 µg·mL^−1^) plus and minus BafA (100 nm) for 18h (*n* = 6). (F) Densitometry of RIPK1 in samples stimulated as shown in (E) (*n* = 6). *P < 0.05, **P < 0.01, ***P < 0.001 was determined using one‐sample t test versus hypothetical value of 100% (in A,C) or by a one‐way ANOVA with Sidak’s *post hoc* comparison (F). Values shown are the mean ± SEM.

These data so far would logically point to an inhibition of lysosomal activity or pH as being responsible for the effects of bafilomycin A1 on the NLRP3 inflammasome. Indeed addition of bafilomycin A1 abolished lysosomal activity in CD14+ monocytes as measured by DQ‐BSA Red cleavage, a fluorogenic substrate which measures lysosomal protease activity, confirming a blockage of pH dependent lysosomal function (Fig. 4A). Bafilomycin A1 inhibited lysosomal protease activity in both the absence and presence of LPS (Fig. 4A). There was also a decrease in lysosomal protease activity after LPS treatment alone (Fig. 4A), possibly due to reduced expression of lysosomal enzymes [23]. To explore this further, we treated primary human CD14+ monocytes with the lysomotropic base NH_4_Cl alongside LPS to directly raise lysosomal luminal pH. Cell lysates were then blotted for active caspase‐1 and lipidated LC3B, and DQ‐BSA fluorescence was monitored 18 h after stimulation. Treatment with 5 mm NH_4_Cl appeared to inhibit lysosomal activity as suggested by an increase in lipidated LC3B (Fig. 4B), but surprisingly there was no effect on DQ‐BSA fluorescence, suggesting that V‐ATPases could still restore lysosomal acidity over a long stimulation period (Fig. 4C). Importantly, there was no effect on LPS‐induced caspase‐1 activity (Fig. 4B). We then tested other disruptors of lysosomal activity. Chloride levels profoundly influence lysosomal function and the broad spectrum Cl^−^ channel inhibitor NPPB is known to modulate lysosomal activity via altering the lysosome luminal Cl^−^ [24]. NPPB (50 µm) strongly inhibited the lysosomal DQ‐BSA signal (Fig. 4C), but also inhibited flux through the autophagy pathway since there was no enhancement of lipidated LC3B in contrast to what we observed with other lysosome disrupting agents (Fig. 4D). Mutations in Niemann–Pick type C 1 (NPC1) cause lysosomal storage disease due to defective lysosomal cholesterol export and can be chemically mimicked by U18666A [25, 26]. Treatment of primary human CD14+ monocytes with U18666A (20 µm) plus LPS inhibited lysosomal activity shown by a significant reduction in DQ‐BSA fluorescence (Fig. 4C) and by an increase in lipidated LC3B (Fig. 4D). However, neither NPPB, nor U18666A treatment enhanced LPS‐induced caspase‐1 activation induced by extracellular LPS in human monocytes (Fig. 4D), although they were previously described to inhibit canonical NLRP3 inflammasome activation [27, 28]. These data suggest that the effects of bafilomycin A1 on the NLRP3 pathway occur independent of lysosomal pH.

Unlike the canonical NLRP3 pathway, the alternative NLRP3 inflammasome pathway is dependent on a protein complex consisting of RIPK1‐FADD‐caspase‐8 upstream of NLRP3 [8]. Since these experiments involved prolonged treatment (18 h) with LPS and bafilomycin A1, we examined the expression levels of RIPK1, FADD, and caspase‐8, as well as components of the NLRP3 inflammasome, to test if effects on expression of these regulators could explain the effects of bafilomycin A1. Neither treatment with LPS or bafilomycin A1 altered the expression of ASC, caspase‐1, caspase‐8 or FADD (Fig. 4E). LPS induced the expression of NLRP3, but this was unaffected by co‐treatment of bafilomycin A1 (Fig. 4E). Levels of RIPK1 were increased by LPS alone and were further enhanced by bafilomycin A1 co‐treatment (Fig. 4E,F). These data suggest the possibility that bafilomycin A1 may potentiate the upstream RIPK1‐FADD‐caspase‐8 signalling leading to enhanced activation of NLRP3.

## Discussion

Understanding the regulation of the NLRP3 inflammasome has attracted widespread interest, in part, due to its contribution to disease [2]. However, understanding NLRP3 is complicated due to the multiple pathways regulating its activity. Proposed pathways include the canonical, the noncanonical, and the alternative NLRP3 pathways [2]. The alternative NLRP3 pathway has been described in primary human monocytes and is characterized by an independence from K^+^ efflux and ASC speck formation [8]. As innate immune cells in the human blood, monocytes possess a unique capacity to activate the alternative inflammasome and secrete IL‐1β in response to extracellular LPS. The levels of IL‐1β released by primary monocytes in response to LPS alone are relatively small compared to the classical two step activation pathway [7], but could circulate and act systemically to modulate disease progression [29]. Here we report that the V‐ATPase may be an important regulator of the NLRP3 inflammasome in primary human monocytes. The specific V‐ATPase inhibitor bafilomycin A1 has been used previously to inhibit canonical two step NLRP3 activation caused by lysosomal dysfunction and particulate matter [8, 30, 31]. Here we demonstrated that inhibition of V‐ATPase activity with bafilomycin A1 led to the exacerbation of NLRP3 inflammasome activation in human monocytes in response to LPS. Thus these data suggested regulation of the NLRP3 pathway by the V‐ATPase.

The data presented here strongly suggest that the V‐ATPase acted independently of lysosomal pH in regulating the NLRP3 inflammasome and point to a regulation of RIPK1. In addition to lysosomal pH, the V‐ATPase is also reported to be important for nutrient sensing, including glucose and amino acids, by mTOR (mammalian target of rapamycin) and AMP‐activated protein kinase which happens at the cytosolic surface of lysosomes [13, 32]. Understanding how V‐ATPase and RIPK1 interact is a subject for future study.

Several sub‐units of the V‐ATPase have been shown to be specifically targeted by pathogenic effectors to enhance the intracellular survival of bacteria [33, 34, 35]. Therefore, enhanced NLRP3 activation in monocytes by V‐ATPase inhibition may represent an enhanced host‐response to certain intracellular pathogens. Whether pathogen infection synergistically alters the monocyte inflammasome responses in human sepsis by endotoxin and V‐ATPase inhibition requires further investigation.

In summary, we report the discovery that the V‐ATPase is an important regulator of the NLRP3 inflammasome pathway in human monocytes. This discovery furthers our understanding of NLRP3 biology and is an important consideration for the development of NLRP3 therapeutics, and for the informed use of existing therapeutic strategies.

## Materials and methods

### Antibodies and reagents

Inflammasome activators used include: LPS from *Escherichia coli* O26:B6 (L2654; Sigma, Poole, UK), nigericin sodium salt (N7143; Sigma), synthetic Pam3CSK4 (tlrl‐pms; Invivogen, Toulouse, France), PGN from *S. aureus* (77140; Sigma), and recombinant human Apo‐ SAA (300‐13; Peprotech, London, UK). Pharmacological agents used include: Bafilomyicin A1 (B1793; Sigma), Concanamycin A (B2091, Axxora, Exeter, UK), MCC950 (PZ0280; Sigma), biotin‐YVAD‐cmk (bYVAD) (AS‐60841; Anaspec, Fremont, CA, USA), NPPB (0593; Tocris, Bristol, UK), U18666A (U3633; Sigma), DQ‐BSA Red (D12051; Thermo Fisher, Waltham, MA, USA), and DMSO (472301; Sigma). Cell‐permeable Control and TRIF blocking peptide were obtained from Invivogen (tlrl‐pitrif). Primary antibodies were used targeting: NLRP3 (Cryo2, AG‐20B‐0014‐C100; Adipogen, San Diego, CA, USA), human IL‐1β (AF‐201; R&D, Abingdon, UK), ASC (AL177, AG‐25B‐0006‐C100; Adipogen), Caspase‐1 p45 and p10 EPR16883 (ab179515; Abcam, Cambridge, UK), human mature Caspase‐1 p20 D7F10 (#3866; CST), IL‐1β (AF‐201‐NA; R&D), LC3B (ab48394; Abcam), Caspase‐8 (ab108333; Abcam), RIPK1 (#3493; Cell Signaling Technology, Waltham, MA, USA), FADD (#2782; Cell Signaling Technology) and HRP‐β‐actin (A3854; Sigma).

### Cell culture and stimulation

Human PBMCs were obtained with consent from healthy human donors (National Health Service Blood and Transplant, Manchester, UK), with full ethical approval from the University Research Ethics Committee (UREC) at the University of Manchester (ref. 2017‐2551‐3945). Briefly, fresh human PBMCs were separated from leukocyte cones using Ficoll reagent (Thermo Fisher) at 400 ***g*** for 40 min at RT with zero deceleration. PBMCs were washed three times with filtered PBS containing 0.5% BSA and 2 mm EDTA (MACs buffer). Human CD14 monocytes were then positively selected from PBMCs using magnetic CD14 MicroBeads and LS columns (Miltenyi Biotec, Woking, UK) according to the manufacturer’s instructions. PBMCs and CD14 + monocytes were counted before being resuspended in RPMI‐1640 medium (Sigma) supplemented with 1% (vol/vol) FBS, 100 U·mL^−1^ penicillin, 100 μg·mL^−1^ streptomycin and 2 mm l‐glutamine.

Human wild‐type (WT) and NLRP3 KO THP‐1 cells were cultured in RPMI‐1640 medium supplemented with 10% (vol/vol) FBS, 100 U·mL^−1^ penicillin, and 100 μg·mL^−1^ streptomycin and 2 mm l‐glutamine. For THP‐1 cell stimulation, suspension cells were counted and resuspended in RPMI‐1640 containing 1% (vol/vol) FBS. For differentiation of the THP‐1 cells, THP‐1 monocytes were seeded at 1 × 10^6^ cells per mL on flat‐bottom plates in the presence of PMA (250 nm for 20 h). PMA‐differentiated THP‐1 cells were then stimulated with indicated treatments. Cultured THP‐1 cells between passages 3–15 were used for experiments.

For inflammasome stimulation, cells were stimulated with 1 µg·mL^−1^ LPS in the presence or absence of drug with DMSO vehicle control for 18 h. For human IL‐1β ELISA and LDH release, culture plates containing monocytes were placed on ice immediately after stimulation and centrifuged at 600 ***g*** for 5 min at 4 °C. Cell supernatants were collected and analysed using human IL‐1β ELISA kit (R&D) and CytoTox 96 cytotoxicity assay (Promega, Southampton, UK) on a Synergy HT plate reader (BioTek, Winooski, VT, USA). For TRIF inhibition experiments, primary human monocytes were incubated with Control or TRIF blocking peptide (50 μm) for 2 h before stimulation. R&D ELISA kits were also used to quantify levels of IL‐1α, IL‐6, and TNF‐α according to manufacturer’s instructions. For total protein western blotting, both cell supernatant and lysates (total lysates) were collected and lysed in 5× SDS‐containing Laemmli buffer and heated at 95 °C for 5 min.

### Western blotting

Total cell lysates were separated by standard Tris‐glycine SDS/PAGE and then transferred onto Polyvinylidene fluoride membranes (Millipore, Watford, UK) at 25 V using a semidry Trans‐Blot Turbo system (Bio‐Rad). Membranes were blocked in 2.5% (wt/vol) BSA or 5% skimmed milk in PBS containing 0.1% (vol/vol) Tween 20 (PBST) before overnight incubation at 4 °C with indicated primary antibodies. Membranes were then labelled with HRP‐conjugated secondary antibodies (Dako, Stockport, UK) and visualized with Amersham ECL prime detection reagent (GE Healthcare, Amersham, UK). Western blot images were captured digitally using a G:Box Chemi XX6 (Syngene, Cambridge, UK). Densitometry was performed against the corresponding β‐actin blot using fiji (Image J, NIH, Bethesda, MD, USA).

### ASC oligomerization assay

Human primary CD14+ monocytes, or WT and NLRP3 KO THP‐1 cells were seeded into 12‐well plates at a density of 1 × 10^6^ cells per mL. Following LPS stimulation (1 µg·mL^−1^, 18 h), cells were directly lysed in the well by the addition of 1% (vol/vol) Nonidet P‐40 (NP‐40) or Triton X100 with 1× protease inhibitor cocktail (Millipore). Cell lysates were separated into NP‐40 or Triton ×100 soluble fraction and insoluble fraction using differential centrifugation at 6800 ***g*** for 20 min at 4 °C. The soluble fraction of cell lysates was subsequently boiled in 1X Laemmli buffer and used for western blotting, whereas the NP‐40 or Triton ×100 insoluble pellets were chemically cross‐linked with 2 mm DSS (Thermo Fisher) in PBS for 30 min at RT. DSS‐cross‐linked pellets were spun down at 6800 ***g*** for 20 min and then heated at 95 °C for 5 min in 1× Laemmli buffer for SDS/PAGE.

### Immunofluorescence

For immunostaining, CD14+ monocytes were adhered to sterilized 13 mm thick glass coverslips at a density of 1 × 10^6^ cells per mL using serum‐free RPMI and incubated at 37 °C for 15 min in 5% CO_2_ [36]. After removing serum‐free RPMI, adherent cells were then stimulated in 1% (vol/vol) FBS RPMI‐1640 medium containing respective treatments for 18 h. Cells were washed with cold PBS once and fixed with 4% (wt/vol) paraformaldehyde for 20 min. Fixed cells were blocked and permeabilized with 1% (wt/vol) BSA, 0.1% (vol/vol) Triton X‐100, and 0.05% (vol/vol) Tween 20 in PBS. Anti‐ASC antibodies (Adipogen) were incubated in 1% (wt/vol) BSA, 0.3% (vol/vol) Triton X‐100 in PBS overnight at 4 °C. Coverslips were then washed three times with PBS before incubation with anti‐rabbit AlexaFluor 594 secondary antibodies (Thermo Fisher) for 1 h at RT. After a further three washes with PBS, cells were stained with 1 µg·mL^−1^ DAPI. Samples were washed three times before being mounted in Prolong Gold (Thermo Fisher). Images were captured using a 20× objective on a Zeiss Axioimager D2 upright microscope (Oberkochen, Germany) and subsequently processed and analysed using imagej software.

### Lysosomal activity

Freshly isolated human CD14+ monocytes were resuspended in serum‐free RPMI and seeded in 96‐well clear bottom plates (Greiner Bio‐One, Stonehouse, UK) at 10^6^ cells per mL for 15 min at 37 °C in 5% CO_2_. Serum‐free RPMI was replaced with 1% (vol/vol) FBS RPMI‐1640 supplemented with or without 10 µg·mL^−1^ DQ‐BSA Red (Thermo Fisher). Cells were stimulated for 18 h with indicated treatments and imaged live using a 20× objective on an Incucyte ZOOM System (Essen BioScience, Royston, UK). Absorbance at 620 nm emission wavelength per field of view was quantified, and absorbance threshold was set using no DQ‐BSA control.

### Quantification and Statistical analysis

Human donors are represented in individual data points. *n* represents experiments performed on individual human donors or different passage THP‐1 cells per independent experiment. Data are presented as mean values ± SEM. Equal variance and normality were assessed with Levene's test and the Shapiro–Wilk test, respectively, and appropriate transformations were applied when necessary. Statistical analyses were carried out using graphpad prism 8 (San Diego, CA, USA). Data with multiple groups were analysed using matched one‐way or two‐way ANOVA followed by Sidak’s *post hoc* analysis. Accepted levels of significance were *P < 0.05, **P < 0.01, ***P < 0.001 and ****P < 0.0001.

## Conflict of interest

The authors declare no conflict of interest.

## Author contributions

SY, JG, GLC and DB planned experiments. SY, JG and RW performed experiments. SY, JG, RW, GLC and DB, analysed data. GLC and DB contributed reagents or other essential material; and SY, JG, RW, GLC and DB wrote the paper.
